# Aplastic anemia associated with interferon alpha 2a in a patient with chronic hepatitis C virus infection: a case report

**DOI:** 10.1186/1752-1947-4-268

**Published:** 2010-08-12

**Authors:** Savvas Ioannou, Gregorios Hatzis, Ioanna Vlahadami, Michael Voulgarelis

**Affiliations:** 1 Department of Pathophysiology, Medical School, National University of Athens, Athens, Greece

## Abstract

**Introduction:**

Hepatitis-associated aplastic anemia is a common syndrome in patients with bone marrow failure. However, hepatitis-associated aplastic anemia is an immune-mediated disease that does not appear to be caused by any of the known hepatitis viruses including hepatitis C virus. In addition, to the best of our knowledge there are no reported cases of patients with chronic hepatitis C virus infection developing aplastic anemia associated with pegylated interferon alpha 2a treatment.

**Case presentation:**

We report the case of a 46-year-old Greek man who developed severe aplastic anemia during treatment with pegylated interferon alpha 2a for chronic hepatitis C virus infection. He presented with generalized purpura and bruising, as well as pallor of the skin and mucous membranes. His blood tests showed pancytopenia. He underwent allogeneic bone marrow transplantation after completing two courses of immunosuppressive therapy with antithymocyte globulin and cyclosporin A.

**Conclusions:**

The combination of a specific environmental precipitant represented by the hepatitis C virus infection, an altered metabolic detoxification pathway due to treatment with pegylated interferon alpha 2a and a facilitating genetic background such as polymorphism in metabolic detoxification pathways and specific human leukocyte antigen genes possibly conspired synergistically in the development of aplastic anemia in this patient. Our case clearly shows that the causative role of pegylated interferon alpha 2a in the development of aplastic anemia must not be ignored.

## Introduction

Hepatitis C virus (HCV) infection is a major public health issue. In developed countries, HCV accounts for 20% of cases of acute hepatitis, 70% of cases of chronic hepatitis, 40% of cases of end-stage cirrhosis, 60% of cases of hepatocellular carcinoma, and 30% of liver transplants [1]. Moreover, extrahepatic manifestations of chronic HCV infection are clinically present in almost 40% of infected patients. These manifestations include essential mixed cryoglobulinemia, sicca syndrome, membranoproliferative glomerulonephritis, thrombocytopenia, and autoimmune hemolytic anemia (AIHA) [2].

Hepatitis-associated aplastic anemia (HAA) is a not uncommon syndrome in patients with bone marrow failure, with hepatitis documented in 2 to 5% of cases of aplastic anemia (AA) occurring in the West [3,4] and 4 to 10% in the Far East [5]. Characteristically, the HAA syndrome is more prevalent among young men. The hepatitis generally follows a benign course, but the onset of AA two to three months later is usually fatal if left untreated. HAA may be induced by the presence of HCV or hepatitis B virus infection, and also by infections with other viruses such as human immunodeficiency virus (HIV), Epstein-Barr virus (EBV), transfusion-transmitted virus and echovirus [6]. However, most cases of HAA are seronegative for the known hepatitis viruses, including hepatitis A, B, C, and G (GB virus C) [7]. The clinical features of the syndrome and the patient's response to immunosuppressive treatment strongly indicate that the liver and marrow abnormalities in patients with HAA are immune-mediated [8,9].

Pegylated interferon alpha 2a (PEG-IFN-α 2a) or 2b plus ribavirin is currently the standard regimen for patients with HCV infection. A wide range of adverse reactions, including flu-like symptoms, nausea, anorexia, diarrhea, psychiatric symptoms, alopecia, injection-site reactions, leukopenia, thrombocytopenia, hemolytic anemia, cough, dyspnea, rash, pruritus, insomnia, and ataxia, have been associated with PEG-IFN-α 2a plus ribavirin treatment. Treatment with interferon (IFN)-α has also been reported to trigger autoimmune phenomena in up to 3% of cases, with AIHA being the most prevalent and most significant phenomena seen in clinical practice [10]. Furthermore, due to its inhibition of cellular growth, interference with oncogene expression and augmentation of lymphocyte cytotoxicity for target cells, IFN-α may cause bone marrow suppression, including potentially severe cytopenias and, very rarely, AA [11].

The primary observed serious adverse side effect of ribavirin treatment is hemolytic anemia. Ribavirin is an antiviral nucleoside analogue; the mechanism of ribavirin-induced hemolytic anemia has not been clearly established. Anemia is most likely related to extensive ribavirin accumulation in erythrocytes subsequent to active unidirectional transmembraneous transport. Ribavirin exerts its toxicity through an inhibition of intracellular energy metabolism and oxidative membrane damage, leading to an accelerated extravascular hemolysis by the reticulo-endothelial system [12]. Lau *et al*. describe how ribavirin, following uptake into cells, is phosphorylated and converted to ribavirin triphosphates, which then must be dephosphorylated for elimination from the cells [13]. However, because red blood cells lack dephosphorylation enzymes, ribavirin accumulates in cells and destroys them, causing hemolytic anemia. Severe anemia develops in about 10% of patients treated with ribavirin, and they require close monitoring of hemoglobin (Hb) levels and often ribavirin dose reduction, which may compromise sustained virologic response.

Herein, we report the development of AA in a patient with chronic HCV infection following treatment with PEG-IFN-α 2a plus ribavirin. By reviewing the literature on the subject and the course of the patient's disease, we have come to the conclusion that, on balance, the development of AA was a side effect of the patient's treatment with PEG-IFN-α 2a within a facilitating genetic and environmental background.

## Case presentation

**Figure 1:**
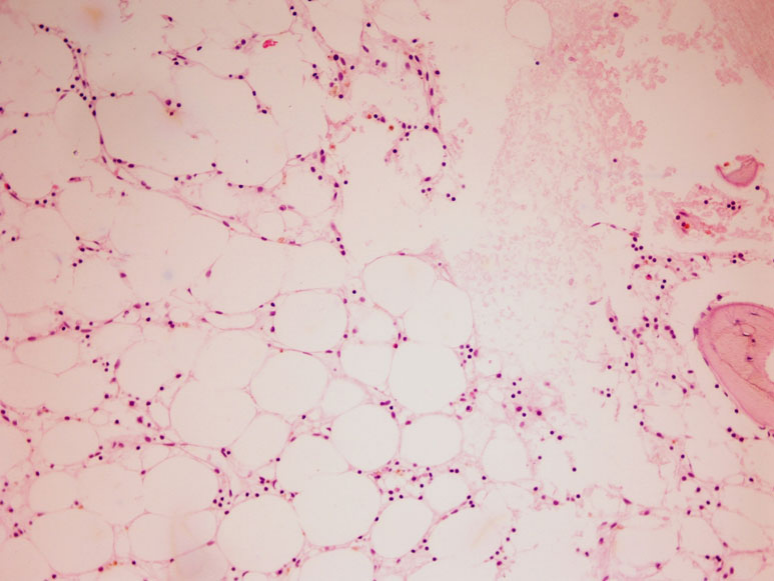
**A bone marrow biopsy showing the absence of hematopoietic tissue and its replacement with fat**. Hematoxylin & eosin staining. 20× magnification.

A 46-year-old Greek man was diagnosed with HCV infection (genotype 4 h) and a combination treatment of PEG-IFN-α 2a (180 μg, weekly) and ribavirin (1200 mg/day) was commenced for a period of 48 weeks. Before starting the combination treatment his blood tests were normal with a platelet count of 250,000 cells/mm^3^, Hb of 16.3 g/dl, and a white blood cell (WBC) count of 6300 cells/mm^3^. The treatment was well tolerated by the patient with a normalization of his liver function tests. Four months later he was referred to the department of pathophysiology with a bleeding tendency and unexplained fatigue of recent onset. No contact with a benzene or pesticide was mentioned by the patient. A physical examination revealed generalized purpura and bruising, and pallor of the skin and mucous membranes. The patient's liver, spleen and lymph nodes were not enlarged. Routine blood work showed severe pancytopenia with a platelet count of 20,000 cells/mm^3^, Hb of 7.9 g/dl, reticulocytes at 0% and a WBC count of 600 cells/mm^3 ^with an absolute neutrophil count of 180 cells/mm^3^. Further investigation showed the patient had a normal liver function test and normal prothrombin time. On admission, his serum HCV ribonucleic acid (RNA) levels were more than 1 × 10^6 ^units/ml. Serology for HIV, and hepatitis A and B viruses was negative, as were immunoglobulin (Ig) M antibodies against cytomegalovirus, parvovirus B19, herpes simplex viruses 1 and 2, and EBV. Further investigations showed the following: urea 20 mg/dl (normal range 17 to 50 mg/dl), creatinine 1.0 mg/dl (normal range 0.7 to 1.4 mg/dl), sodium 139 mMol/L (normal range 136 to 145 mMol/L), potassium 3.8 mMol/L (normal range 3.5 to 5.0 mMol/L), glucose 99 mg/dl (normal range 74 to 115 mg/dl), calcium 8.8 mg/dl (normal range 8.6 to 10.2 mg/dl), amylase 48 U/L (normal range 20 to 104 U/L), creatine phosphokinase 200 U/L (normal range 20 to 190 U/L), lactate dehydrogenase 296 U/L (normal range 200 to 460 U/L), uric acid 4.6 mg/dl (normal range 3.5 to 7.2 mg/dl), erythrocyte sedimentation rate 34 mm in the first hour (normal range 0 to 20 mm), and C-reactive protein 37.4 mg/L (normal range 0 to 5 mg/L). Screening for several autoantibodies was negative. Thyroid function tests and complement serum levels were normal. A serum protein electrophoresis showed no hypogammaglobulinemia or abnormal bands. A computed tomography examination of the patient's abdomen and thorax was unremarkable. The patient's bone marrow biopsy was profoundly hypocellular with a decrease in all haematopoietic cells (Figure 1); the bone marrow space was composed mostly of fat cells and marrow stroma. The CD34 cell population was more than 1%. Malignant infiltrates or fibrosis were absent. Fluorescence-activated cell sorting analysis of the patient's bone marrow showed decreased marrow elements with normal lymphocyte gate. A cytogenetic examination showed the patient had a normal karyotype. The presence of paroxysmal nocturnal hemoglobulinuria was excluded by flow cytometry with the use of anti-CD55 and anti-CD59 antibodies. Human leukocyte antigen (HLA) typing revealed the presence of DRB1*0701 and DRB1*1501 alleles. HLA matching identified a sister with an identical HLA type.

The diagnosis of severe AA was made in the patient. Treatment with PEG-IFN-α 2a and ribavirin were discontinued. However, after two weeks, the pancytopenia did not resolve and the patient was started on immunosuppressive therapy with rabbit antithymocyte globulin (Thymo-globulin, Genzyme; 15 mg/kg/day, for five consecutive days) and cyclosporin A (6 mg/kg/day, in divided doses every 12 hours). Prophylaxis against serum sickness was instituted with methylprednisolone (2 mg/kg/day) for five days with subsequent halving of the dose every week until discontinuation on day 28. The patient had a partial response that was noted on day 60 with a platelet count of 27,000 cells/mm^3^, Hb of 9.3 g/dl and WBC of 5000 cells/mm^3^. The patient was dependent on red blood cell and platelet transfusions and was on granulocyte colony stimulating factor (400 μg/m^2^/day, three times a week). Therefore, on day 120 a second course of antithymocyte globulin therapy was given. The patient received a full cyclosporin A dose for six months, after which cyclosporin A was tapered off slowly (0.5 mg/kg/month). During the period of aplasia, the patient was persistently pyrexial and broadspectrum antibiotics in the form of an antipseudomonal penicillin (piperacillin/tazobactam) and a carbapenem (meropenem) were administered consecutively, as well as an antifungal agent (liposomal amphotericin B).

Eight months after the first course of immunosuppressive treatment, the patient's Hb was 10.6 g/dl, platelet count was 32,000 cells/mm^3 ^and WBC was 3590 cells/mm^3 ^with an absolute neutrophil count of 2261 cells/mm^3^. At that time the patient was still receiving blood and platelet transfusions. His serum HCV RNA levels were more than 1 × 10^6 ^units/ml indicating that the patient was continuously viremic. His liver function tests remained normal during follow up. The patient underwent allogeneic bone marrow transplantation. He experienced a hemorrhagic stroke due to prolonged thrombocytopenia and died during the recovery phase.

## Discussion

AA is characterized by a diminished number of or absent bone marrow precursor cells and peripheral cytopenias. The disease is estimated to occur in two to four people per million per year [14,15]. Numerous studies have shown that AA behaves as an immune-mediated disease. Cytotoxic T cells expressing T-helper 1 cytokines, especially IFN-γ, have been implicated in the pathophysiology of T cell-induced, Fas-mediated stem cell apoptosis of CD34 target stem cells [16]. Why T cells are activated in patients with AA is unclear. A number of reports have documented a significantly increased incidence of HLA-DR15 in patients with AA [17]. Additionally, in a recent study, HLA-DRB1 gene analysis showed an increased prevalence of DRB1*07 in patients with AA compared with the normal population, at 15.7% and 8.3%, respectively. This raises the possibility that HLA-DRB1*07 plays a significant role in the development of AA [18]. Our patient had both DRB1*0701 and DRB1*1501 alleles, which may indicate that their presence is likely to allow for preferential presentation of peptides, such as viruses or drugs, to specific T cells, driving the autoimmune T cell-mediated destruction of the patient's hematopoietic cells. This process might have been further enhanced both in quantity and quality by the action of the IFN-α treatment that the patient received.

However, the association of AA and chronic HCV infection remains ill-defined. In a recent report, there were two cases of patients with AA, unrelated to IFN-α therapy, among 35 patients with chronic HCV infection [19]. Another case of a patient with severe AA associated with HCV infection has also been reported [20]. Several other studies have shown that the prevalence of anti-HCV antibodies in patients with HAA receiving blood transfusions increases with the duration and number of transfusions, and is therefore probably transfusion related [21]. Taking these data into account and considering our patient's clinical course (normal liver function tests at presentation, late onset of AA), it is unlikely that the HCV infection alone was the cause of his ensuing AA.

Bone marrow aplasia may also occur as an idiosyncratic drug reaction, with a sudden onset after several months of therapy, and it is usually irreversible. In this regard, two cases of patients with bone marrow hypoplasia and fibrosis following IFN-α treatment have been reported in the literature [22]. A severe and persistent pancytopenia has also been described in a 42-year-old woman with a non-Hodgkin's lymphoma following a course of 10 days of intramuscular leukocyte IFN-α [23]. Aslam and Singh reported a case of AA with IFN-β 1a in a patient with multiple sclerosis [24]. However, to date, there have been no reports of patients with severe AA associated with PEG-IFN-α 2a in chronic HCV infection.

Some reports have suggested a genetic predisposition to bone marrow injury in patients with an idiosyncratic drug reaction. In such cases, direct toxicity may occur, possibly due to genetically determined differences in metabolic detoxification pathways [25,26]. Interestingly, the most commonly used dose of IFN-α in humans inhibits cytochrome P450, thus decreasing the hepatic clearance of some drugs, and this inhibition persists during IFN-α therapy leading to various forms of hepatic and extrahepatic toxicity [27].

On the other hand, clinical characteristics and circumstantial evidence suggest that idiosyncratic drug reactions are caused by reactive metabolites and are immune-mediated. The possible mechanisms of stem cell damage by drug-mediated immune damage have not been clearly defined. One suggestion mechanism is the 'spoiled membrane hypothesis', which envisages aberrant stem cell antigens as a result of drug action [28]. Another possibility that has not been widely explored is that drug-induced AA is uncommon because it requires a coincident event at or near the time the drug is given. We could speculate that such an event might be a virus infection such as with HCV. Therefore, we suggest that the combination of a specific environmental precipitant represented by the HCV infection, an aberrant expression of cellular proteins in the patient's bone marrow cells caused by a disturbed PEG-IFN-α 2a-associated drug metabolic detoxification pathway, and a facilitating genetic background (specific HLA genes) offering a more effective presentation of viral and drug metabolites to the T cells conspired, possibly synergistically, in the initiation of the destructive immune attack towards the patient's bone marrow cells and the development of severe AA in our patient.

The approach to treating a patient with medication-induced AA entails stopping the offending drug while supporting the patient during the period of pancytopenia. The therapeutic issue revolves around the dilemma of a period of initial observation versus aggressive therapy, such as immunosuppression or bone marrow transplantation. Waiting for a week and then conducting a repeat bone marrow biopsy may avoid potential side effects associated with the therapy without foreclosing on a definitive treatment, which is to be promptly instituted in the absence of signs of recovery.

## Conclusions

We present a case of a 46-year-old man who developed severe AA while being treated with PEG-IFN-α 2a for chronic HCV infection. To the best of our knowledge, this is the first report of a patient with this complication associated with PEG-IFN-α 2a in the growing body of literature. As health care providers, physicians should be aware of this rare but life-threatening complication of PEG-IFN-α 2a treatment.

## Abbreviations

AA: aplastic anemia; AIHA: autoimmune hemolytic anemia; EBV: Epstein-Barr virus; HAA: hepatitis-associated aplastic anemia; Hb: hemoglobin; HCV: hepatitis C virus; HIV: human immunodeficiency virus; HLA: human leukocyte antigen; IFN: interferon; Ig: immunoglobulin; PEG-IFN-α 2a: pegylated interferon alpha 2a; RNA: ribonucleic acid; WBC: white blood cells.

## Consent

Written informed consent was obtained from the patient for publication of this case report and any accompanying images. A copy of the written consent is available for review by the Editor-in-Chief of this journal.

## Competing interests

The authors declare that they have no competing interests.

## Authors' contributions

SI and MV were responsible for writing the manuscript. IV and GH provided clinical details and contributed to the final manuscript. All authors read and approved the final manuscript.

## References

[B1] EASL International Consensus Conference on Hepatitis C Consensus statement J Hepatol 1999 30 956 961 10.1016/S0168-8278(99)80154-8 10365827

[B2] Palekar NA Harrison SA Extrahepatic manifestations of hepatitis C South Med J 2005 98 1019 1023 10.1097/01.smj.0000182873.62872.22 16295816

[B3] Bottiger LE Westerholm B Aplastic anaemia. III. Aplastic anaemia and infectious hepatitis Acta Med Scand 1972 192 323 6 5081070

[B4] Mary JY Baumelou E Guiguet M Epidemiology of aplastic anemia in France: A prospective multicentric study Blood 1990 75 1646 53 2183887

[B5] Young NS Issaragrasil S Chieh CW Takaku F Aplastic anaemia in the Orient Br J Haematol 1986 62 1 6 10.1111/j.1365-2141.1986.tb02893.x 3942690

[B6] Gonzalez-Casas R Jones EA Moreno-Otero R Spectrum of anemia associated with chronic liver disease World J Gastroenterol 2009 15 4653 4658 10.3748/wjg.15.4653 19787828 PMC2754513

[B7] Brown KE Tisdale J Barrett AJ Dunbar CE Young NS Hepatitis-associated aplastic anemia N Engl J Med 1997 336 1059 1064 10.1056/NEJM199704103361504 9091802

[B8] Gonzalez-Casas R Garcia-Buey L Jones EA Gisbert JP Moreno-Otero R Systematic review: hepatitis-associated aplastic anaemia-a syndrome associated with abnormal immunological function Aliment Pharmacol Ther 2009 30 436 443 10.1111/j.1365-2036.2009.04060.x 19508613

[B9] Lu J Basu A Melenhorst JJ Young NS Brown KE Analysis of T-cell repertoire in hepatitis-associated aplastic anemia Blood 2004 103 4588 4593 10.1182/blood-2003-11-3959 14988156

[B10] Conrad B Potential mechanisms of interferon-alpha induced autoimmunity Autoimmunity 2003 36 519 523 10.1080/08916930310001602137 14984029

[B11] Platanias LC Fish EN Signaling pathways activated by interferons Exp Hematol 1999 27 1583 1592 10.1016/S0301-472X(99)00109-5 10560905

[B12] Russmann S Grattagliano I Portincasa P Palmieri VO Palasciano G Ribavirin-induced anemia: mechanisms, risk factors and related targets for future research Curr Med Chem 2006 13 3351 3357 10.2174/092986706778773059 17168855

[B13] Lau JY Tam RC Liang TJ Hong Z Mechanism of action of ribavirin in the combination treatment of chronic HCV infection Hepatology 2002 35 1002 1009 10.1053/jhep.2002.32672 11981750

[B14] Young NS Acquired aplastic anemia Ann Intern Med 2002 136 534 46 11926789 10.7326/0003-4819-136-7-200204020-00011

[B15] Wallerstein RO Condit PK Kasper CK Statewide study of chloramphenicol therapy and fatal aplastic anemia JAMA 1969 208 2045 50 10.1001/jama.208.11.2045 5818983

[B16] Young NS Calado RT Scheinberg P Current concepts in the pathophysiology and treatment of aplastic anemia Blood 2006 108 2509 2519 10.1182/blood-2006-03-010777 16778145 PMC1895575

[B17] Sugimori C Yamazaki H Feng X Mochizuki K Kondo Y Takami A Chuhjo T Kimura A Teramura M Mizoguchi H Omine M Nakao S Roles of DRB1 *1501 and DRB1 *1502 in the pathogenesis of aplastic anemia Exp Hematol 2007 35 13 20 10.1016/j.exphem.2006.09.002 17198869

[B18] Yari F Sobhani M Vaziri MZ Bagheri N Sabaghi F Talebian A Association of aplastic anaemia and Fanconi's disease with HLA-DRB1 alleles Int J Immunogenet 2008 35 453 456 10.1111/j.1744-313X.2008.00810.x 19046304

[B19] Ramos-Casals M García-Carrasco M López-Medrano F Trejo O Forns X López-Guillermo A Muñoz C Ingelmo M Font J Severe autoimmune cytopenias in treatment-naive hepatitis C virus infection: clinical description of 35 cases Medicine (Baltimore) 2003 82 87 96 10.1097/00005792-200303000-00003 12640185

[B20] Gruber A Grillner L Norder H Magnius L Björkholm M Severe aplastic anemia associated with seronegative community-acquired hepatitis C virus infection Ann Hematol 1993 66 157 159 10.1007/BF01697628 7682449

[B21] Paquette RL Kuramoto K Tran L Sopher G Nimer SD Zeldis JB Hepatitis C virus infection in acquired aplastic anaemia Am J Hematol 1998 58 122 126 10.1002/(SICI)1096-8652(199806)58:2<122::AID-AJH6>3.0.CO;2-U 9625579

[B22] Hoffmann A Kirn E Krueger GR Fischer R Bone marrow hypoplasia and fibrosis following interferon treatment In Vivo 1994 8 605 612 7893989

[B23] Mangan KF Zidar B Shadduck RK Zeigler Z Winkelstein A Interferon-induced aplasia: evidence for T-cell-mediated suppression of hematopoiesis and recovery after treatment with horse antihuman thymocyte globulin Am J Hematol 1985 19 401 413 10.1002/ajh.2830190411 2411129

[B24] Aslam AK Singh T Aplastic anemia associated with interferon beta-1a Am J Ther 2002 9 522 523 10.1097/00045391-200211000-00011 12424511

[B25] Lee KA Kim SH Woo HY Hong YJ Increased frequencies of glutathione S-transferase (GSTM1 and GSTT1) gene deletions in Korean patients with acquired aplastic anemia Blood 2001 98 3483 3485 10.1182/blood.V98.12.3483 11719393

[B26] Poonkuzhali B Shaji RV Salamun DE George B Srivastava A Chandy M Cytochrome P4501A1 and glutathione S transferase gene polymorphisms in patients with aplastic anemia in India Acta Haematol 2005 114 127 132 10.1159/000087885 16227674

[B27] Israel BC Blouin R McIntyre W Shedlofsky S Effects of interferon-? monotherapy on hepatic drug metabolism in cancer patients Br J Pharmac 1993 36 229 235 10.1111/j.1365-2125.1993.tb04222.x PMC1364643 9114909

[B28] Waring JF Anderson MG Idiosyncratic toxicity: mechanistic insights gained from analysis of prior compounds Curr Opin Drug Discov Devel 2005 8 59 65 15679173

